# Addressing malnutrition in Ethiopia: A call for a systems approach to match the scope and complexity of the problem

**DOI:** 10.1111/mcn.13701

**Published:** 2024-07-04

**Authors:** Stanley Chitekwe, Kaleab Baye, Ramadhani Noor, Christiane Rudert

**Affiliations:** ^1^ Nutrition Section UNICEF Ethiopia Addis Ababa Ethiopia; ^2^ Center for Food Science and Nutrition College of Natural and Computational Sciences, Addis Ababa University Addis Ababa Ethiopia; ^3^ Nutrition and Food Systems' Division Research Center for Inclusive Development in Africa (RIDA) Addis Ababa Ethiopia; ^4^ Regional Nutrition Adviser UNICEF Eastern and Southern Africa Regional Office (ESARO) Nairobi Kenya

**Keywords:** malnutrition, nutrition interventions, systems' approach, triple‐burden

## 1 BACKGROUND

Over the past couple of decades, Ethiopia has made tremendous progress in reducing child morbidity and mortality. Child mortality decreased from 140.7 deaths/1000 live births in 2000 to 48.7 deaths/1000 live births in 2020 (United Nations Inter‐agency Group for Child Mortality Estimation, 2021). Stunting has significantly declined, from 57.7% in 2000 to 36.8% in 2019; similarly, the prevalence of wasting decreased from 12.2% to 7.0% during the same period (Central Statistical Authority, 2001; Ethiopian Public Health Institute, 2019). These reductions in malnutrition were primarily attributed to the expansion of access to health care services through the health extension programme, increased income, and reduction in open defecation (Headey et al., 2017). While these improvements are encouraging, much remains to be done as the rates of malnutrition remain high. To sustain but also accelerate progress in preventing malnutrition, there is an urgent call for more effective interventions that match the scope, complexity, and systemic nature of the problem. This supplement entitled ‘Aligning food, health, education, and WASH systems to reduce malnutrition in Ethiopia’ aimed to respond to this call.

Understanding the magnitude and distribution of the problem and identifying the drivers that led to observed changes is the first step toward the design of much‐needed interventions. The 15 articles in this supplement provide a unique diagnosis of the problem of malnutrition in Ethiopia. The supplement highlights the trends, magnitude, and distribution of various forms of malnutrition, highlighting prevailing inequalities, and identifies several drivers. The supplement also presents promising approaches and interventions that could be considered for scale‐up.

## 2 TRENDS, MAGNITUDE, AND DISTRIBUTION OF MALNUTRITION

Using a longitudinal study, Hirvonen et al. (2021a) evaluated the dynamics of child linear and ponderal growth faltering. This is indeed a very important contribution for a country like Ethiopia, where both stunting and wasting remain serious public health concerns. The analyses revealed that the prevalence of child wasting peaks in the first 6 months of life, whereas that of stunting starts only to increase significantly after 6 months of age. This is in line with earlier findings that linked the timing of growth faltering with the complementary feeding period (Victora et al., 2010), but also signifies the beginning of the manifestations of sustained nutritional deprivation and recurrent infections faced in the first months of the child (Benjamin‐Chung et al., 2023). Worth noting is also the high (15%–20%) prevalence of stunting reported to be present at birth; a finding that suggests that poor maternal nutritional status, particularly during pregnancy, is contributing to the high burden of malnutrition. Indeed, the study by Hailu et al. (2021) showed that more than one in five women of reproductive age were anaemic in 2016, but with even higher prevalence (>50%) in some of the identified hotspots. Unlike child nutritional outcomes, the prevalence of anaemia was reported to have increased between the periods of 2000 and 2016, and these increases were primarily reflections of the widening of existing hotspot areas. Such subnational analyses and mapping help to identify priority areas, but also unmask disparities by highlighting areas that have made little or no progress. Similarly, the subnational estimates and analyses helped unravel the increasing problem of the Double Burden of Malnutrition (DBM), defined as the coexistence of undernutrition and overweight/obesity or diet‐related noncommunicable diseases (NCDs), which could be at the individual, household, or population level (WHO, 2016). In cities like Addis Ababa, the prevalence of household‐level DBM was quite high (22.8%), while relying on the national estimates only would have painted a very low prevalence of DBM (3.6%: 2016; Pradeilles et al., 2022). Such high prevalence of DBM in cities like Addis Ababa is not surprising and aligns with a recent multicountry study that related high DBM prevalence among the richest and in the most socially and economically globalized settings, which is the case for major cities like Addis Ababa (Seferidi et al., 2022).

## 3 DRIVERS OF MALNUTRITION AND PREVAILING INEQUALITIES

Several drivers of malnutrition have been identified by the studies published in this supplement. More importantly, the studies identified shared drivers that can help us take the necessary steps to address nutritional problems (e.g., stunting and wasting) through more comprehensive interventions that capture the interrelationships between the different manifestations of malnutrition, rather than focusing on those that address them in isolation.

### 3.1 Diet quality

The paper by Hirvonen et al. (2021a) found that both wasting and stunting were associated with limited consumption of nutrient‐dense foods like animal‐source foods, maternal IYCF knowledge, and increased number of under‐5 children in households. Delayed introduction to complementary feeding was reported to be widespread and associated with gaps in maternal IYCF knowledge, and increased odds of linear growth faltering (Hirvonen et al., 2021b). Progress in improving the dietary diversity of children was too little too slow (Tizazu et al., 2022), and was outpaced by increases in unhealthy feeding practices such as the consumption of ultraprocessed foods (UPFs) (Tizazu et al., 2022b). The increasing consumption of unhealthy foods was also highlighted to be widespread among adolescents in urban schools in Ethiopia, as highlighted by Iyasu et al. (2023) in a qualitative study, also published in this supplement. Worth noting, is also the finding that food safety concerns determine adolescent's food choices and diet quality, underlying the need to integrate food safety considerations into the design of nutrition programmes.

### 3.2 Access and quality of healthcare

Access and quality of health care are critical to prevent excess child death and improve diet quality and nutritional outcomes. The health system is indeed the primary vehicle for the delivery of nutrition‐specific interventions that aim to reduce maternal and child malnutrition. In this supplement, Laillou et al. (2022) illustrate this by estimating the number of child deaths averted between 2008 and 2020 by the Community‐based Management of Acute Malnutrition (CMAM) programme. The CMAM programme was reported to have averted ~34,000 child deaths per year and was reported as one of the cost‐effective ways of preventing death related to child wasting. While appreciating the progress made in the treatment of wasting, the authors call for more efforts to not only treat but prevent wasting. This aligns with UNICEFs call for a systems approach to nutrition, strengthening integration between various systems (e.g. food, water, education, and social protection) (UNICEF, 2020).

Using the co‐coverage index, which is a count of essential reproductive, maternal, neonatal, and child health (RMNCH) interventions received, Baye et al. (2022) showed that access to health care reduces the odds of child wasting, stunting, but also increases dietary diversity. This is supported by the findings from the decomposition study by Girma et al. (2022) and the longitudinal study of Hirvonen et al. (2021a)—both in this supplement—that showed that infections were among the primary predictors of child wasting. The IYCF knowledge of health workers was also found associated with increased maternal IYCF knowledge and MDD, leading the authors to suggest that frequent and timely visits of households by health extension workers to provide messages on what, when, and how to feed the child are needed (Hirvonen et al., 2021b). On the other hand, the finding that IFA supplementation is associated with reduced odds of anaemia among women of reproductive age further suggests the need to strengthen the health system to expand coverage but also provide quality services (Hailu et al., 2021).

### 3.3 Water, sanitation and hygiene (WASH)

Poor WASH conditions can expose vulnerable groups to diarrhoeal infections, compromise the safety of complementary foods, but also influence food choices away from perishable nutrient‐dense foods. The study by Girma et al. (2021) in this supplement showed that improvements in WASH conditions between 2000 and 2016 led to modest reductions in the risk of diarrhoea and stunting in children less than 5 years of age. However, with less than 10% of households having access to basic sanitation facilities, much remains to be done. The timing of diarrhoea coincided with the complementary feeding period (6–23 months), a finding also reported in a multicountry study from Sub‐Saharan Africa (Ogbo et al., 2017). Altogether, this suggests the need to ensure the safety of complementary foods through the implementation of baby WASH interventions (Waller et al., 2020).

## 4 PREVENTING MALNUTRITION: PROMISING INTERVENTIONS AND WHAT COULD BE DONE BETTER?

Socioeconomic well‐being, maternal knowledge/education, diet quality, and access to quality health care were common drivers of all forms of malnutrition. Thus, accelerating progress requires alignment of policies, programmes, and interventions and leveraging synergies across food, health, WASH, and education systems as highlighted in the UNICEF global strategy 2020–2030 (UNICEF, 2020).

First, inequalities in diet, access to health care, and WASH need to be addressed (Girma et al., 2021; Tizazu et al., 2022). According to the studies by Girma et al. (2021) and Tizazu et al. (2022), rural households and those from lower socioeconomic status were those that could not afford to consume nutrient‐dense food groups, access essential health‐ and WASH services; hence, were the most affected by various forms of malnutrition. Increasing income and its distribution, but also boldly investing in women's empowerment could help improve maternal and child well‐being. Indeed, in this supplement, Baye, Laillou, et al. (2021) showed that women empowerment measures (autonomy and decision‐making) were more strongly associated with increased child dietary diversity (MDD) than wealth, child age, and urban residence.

Second, it is critical to intensify efforts to promote the consumption of healthier foods and discourage unhealthy UPFs. However, to succeed these efforts should be accompanied by monitoring and regulations of the food environment, but also bold and innovative interventions that make nutrient‐dense foods available, accessible, and affordable. The article on whole egg powder in this supplement provides an example of such innovations. Using the “cost of diet analyses”, the authors reported that including egg powders into the food, basket helped reduce the minimum‐cost nutritious diet by about 14%, allowing an additional ~1.2 million households to afford the optimized diet (Baye, Abera, et al., 2021). Scalable solutions like these are needed to make other missing nutrient‐dense food groups more accessible and affordable.

Third, increasing coverage but also quality of health and nutrition interventions is critical. Using an end‐user monitoring (EUM) system, the study by Donzé et al. (2022) also in this supplement, illustrates how routine data capture can be facilitated to support timely decision‐making that can improve the delivery of nutrition interventions through the health system. Also key to improving quality and effective coverage of nutrition interventions is to identify the cause of the problem. To this end, efforts to understand the aetiology of anaemia and child wasting are still limited and urgently needed to make much‐needed progress on this front.

Lastly, the interventions in the education system and social protection systems need to be further leveraged to fill existing programme gaps. Empowering children and youth through education, healthier school meals, along with well‐designed social protection programmes that bridge various forms of inequalities can help break the vicious intergenerational cycle of malnutrition (Huicho et al., 2020; Wang et al., 2021). Schools can serve as platforms to promote healthy diets and lifestyles (Iyasu et al., 2023). Targeting adolescents can help shape dietary habits and preferences, having lifelong implications. On the other hand, well‐designed nutrition‐sensitive social protection programmes like cash transfers could help escape the poverty trap and help realize the aspirations of the Sustainable Development Goals of “leaving no one behind” (Renzaho et al., 2019).

Altogether, this supplement provides a unique and comprehensive diagnostic of the problem of malnutrition in Ethiopia. The supplement provides a first attempt to collect evidence supporting the much‐needed transition towards a better alignment of food, health, education, and WASH systems to effectively address all forms of malnutrition in Ethiopia.

## AUTHOR CONTRIBUTIONS

Stanley Chitekwe, Kaleab Baye, Ramadhani Noor and Christiane Rudert had equal roles in drafting and reviewing the manuscript.

## CONFLICT OF INTEREST STATEMENT

The authors declare no conflicts of interest.

## Data Availability

Data sharing is not applicable to this article as no new data were created or analyzed in this study.
